# Towards an Ensemble Vaccine against the Pegivirus Using Computational Modelling Approaches and Its Validation through In Silico Cloning and Immune Simulation

**DOI:** 10.3390/vaccines9080818

**Published:** 2021-07-23

**Authors:** Bowen Zheng, Muhammad Suleman, Zonara Zafar, Syed Shujait Ali, Syed Nouman Nasir, Namra, Zahid Hussain, Muhammad Waseem, Abbas Khan, Fakhrul Hassan, Yanjing Wang, Dongqing Wei

**Affiliations:** 1 School of Life Sciences, Shanxi University, Taiyuan 030006, China; bowen0513@foxmail.com; 2 Center for Biotechnology and Microbiology, University of Swat, Swat 01923, Pakistan; suleman@uswat.edu.pk (M.S.); shujaitswati@uswat.edu.pk (S.S.A.); zahid@uswat.edu.pk (Z.H.); 3 Centre of Agricultural Biochemistry and Biotechnology, University of Agriculture Faisalabad, Faisalabad 38000, Pakistan; zony_bbt@yahoo.com; 4 National Center for Bioinformatics, Quaid-i-Azam University, Islamabad 45320, Pakistan; nnasir@bs.qau.edu.pk; 5 Faculty of Rehabilitation and Allied Health Science, Riphah International University, Islamabad 46000, Pakistan; namraahmad444@icloud.com (N.); wangyanjing@sjtu.edu.cn (Y.W.); dqwei@sjtu.edu.cn (D.W.); 6 Department of Bioinformatics and Biological Statistics, School of Life Sciences and Biotechnology, Shanghai Jiao Tong University, Shanghai 200240, China; m.waseem@riphah.edu.pk; 7 School of Life Science and Technology, University of Electronic Science and Technology of China, Chengdu 610054, China; fakhar_ibneadam@yahoo.com; 8 Peng Cheng Laboratory, Shenzhen 518055, China; 9 State Key Laboratory of Microbial Metabolism, Shanghai-Islamabad-Belgrade Joint Innovation Center on Antibacterial Resistances, Joint Laboratory of International Cooperation in Metabolic and Developmental Sciences, Ministry of Education and School of Life Sciences and Biotechnology, Shanghai Jiao Tong University, Shanghai 200030, China

**Keywords:** Pegivirus, epitopes, vaccine designing, in silico cloning, immune simulation

## Abstract

Pegivirus, HPgV, which was earlier known as Gb virus and hepatitis G virus, is an enveloped, positive-stranded RNA and lymphotropic virus classified into the *Flaviviridae* family. The transmission routes primarily involve blood products, and infections are worldwide, leading up to 25% of persistent infections. To date, no effective therapeutic means are available to clear Pegivirus infections. Effective vaccine therapeutics is the best alternative to manage this disease and any associated potential pandemic. Thus, whole proteome-based mining of immunogenic peptides, i.e., CTL (cytotoxic T lymphocytes), HTL (helper T lymphocytes), and B cell epitopes, was mapped to design a vaccine ensemble. Our investigation revealed that 29 different epitopes impart a critical role in immune response induction, which was also validated by exploring its physiochemical properties and experimental feasibility. In silico expression and host immune simulation were examined using an agent-based modeling approach and confirmed the induction of both primary and secondary immune factors such as IL, cytokines, and antibodies. The current study warrants further lab experiments to demonstrate its efficacy and safety.

## 1. Introduction

Pegivirus, HPgV, which was earlier known as Gb virus and hepatitis G virus, is an enveloped, positive-stranded RNA and lymphotropic virus classified into the *Flaviviridae* family. It has a genome of approximately 94,000 nucleotides. The virus has its replication sites in the bone marrow, thymus, and peripheral blood mononuclear cells (PBMCs). It has infected 750 million people worldwide, with its incidence increasing [1]. The transmission routes primarily involve blood products, and infections are throughout the world, leading up to 25% of persistent infections [1,2]. HPgV codes for the basic proteins enriched with arginine and leucine amino acids. It comprises C, E1, and E2 as structural proteins that are believed to be involved in creating an envelope for the virus and NS3–NS5B as non-structural proteins similar in function to helicase and polymerase proteins of other related viruses of the *Flaviviridae* family [3]. The infection caused by the Pegivirus needs E1 and E2 glycoproteins for cellular attachment to stimulate the infection cascade [4]. The infections are frequently reported in HIV-positive patients. Other studies relate co-infections of HPgV and HIV in aggravating the CD4+ counts [4,5]. Furthermore, HPgV expression was observed in 40% of patients with preceding HIV or HCV infection [6]. Research in Mexico has acquired the prevalence of HIV and HPgV co-infection. Out of 1406 HIV patients, 33% resulted in a broader range expression of viral loads of HPgV through RT-PCR [7]. Recent studies revealed the association of HPgV viremia with a risk of lymphoma, which is the abnormal growth of lymphocytes [8]. The best association with a disease is non-Hodgkin lymphoma (NLH) [9]. Individuals with HPgV viremia have higher risks of establishing large diffuse B-cell, follicular, marginal zone, T-cell lymphomas, Hodgkin lymphoma, and chronic lymphocytic leukemia [8]. Individuals with prior arthritis (RA), primary Sjogren syndrome (pSS), and systemic lupus erythematosus (SLE) pose higher risk of development of non-Hodgkin lymphoma (NHL) [10].

The epidemiological statistics revealed that only 2% of the healthy population is infected with HPgV. Among the transmission routes, vertical, sexual, and blood-borne routes are frequently reported. A prevalence study in the Kyusyu University Hospital between May 1997 and September 2017 revealed HPgV viremia in 2 out of 187 patients (1.1%) with hepatectomy and 44 out of 313 liver transplant recipients (14.1%) [11]. Studies from Southern Europe show the strongest linkage, while North America, Northern Europe, and the Middle East the weakest linkage, with lymphoma risk. [8]. Globally, lymphoid malignancies have caused approximately 6.7 million deaths, according to the Global Burden of Disease Study 2021 [12]. A mortality rate of 8.3 per 100,000 individuals was observed in 2014 in the USA [13,14]. Korea recorded a total of 6638 lymphoid malignancies in 2012, and the incidence rates of all lymphoid malignancies increased from 6.9 to 9.9 per 100,000 persons during the period 1999–2012. [13,15]. HpGV-1 is also reported in association with leukoencephalitis causing inflammatory variation in the cerebral brain. RNA sequences of the virus obtained from the brain resulted in linkages with other Pegiviruses despite deleting 87 nucleotides from the NS2 gene [16]. Cases of early stages for lymphoma are treated by radiation therapy, although patients exhibiting stage 3 and 4 are prescribed chemotherapy, immunotherapy, and radio immunotherapy. Such individuals have high survivorship yet low cure rate [17]. The sole prophylaxis for NHL used to be cytotoxic chemotherapy; however, since 1994 healthy- dose chemotherapy and autologous stem cell transplants have rendered a platform for treatment of aggressive lymphoma, which is too expensive to be afforded by everyone [18]. Diffuse large B-cell lymphoma of stage 1 is managed by a combination of doxorubicin and rituximab chemotherapy, subsequently by radiation therapy [17]. Malignant cases are managed with rituximab and chemotherapy [17]. Follicular lymphoma is reported to have shown recurring progression in short intervals [19]. Since many of the subtypes of lymphoma appear to be incurable with contemporary management approaches, and patients tend to be chemo-resistant due to disease relapse, there is an urgent need to discover efficient prophylaxis [17,20].

Vaccination is one of the most effective and reliable ways to cure a disease and has been reported to prevent 2–3 million deaths per year [21]. It triggers an immune response to the current and future threat of infection [22,23,24]. Since the best approach to control infectious diseases is vaccination, we constructed a multi-epitope subunit vaccine against Pegivirus. It is efficient, inexpensive, less time consuming, and would convincingly overcome the current as well as future catastrophe likely to be brought by lymphoma and its associated ailments. Thus, herein, an immune-informatics pipeline was employed to target the proteome of the Pegivirus to construct an immunogenic epitope (CTL, HTL, and B cell) vaccine ensemble. The current study would help experimental setups to demonstrate its efficacy and safety.

## 2. Materials and Methods

### 2.1. Data Retrieval

**Figure 1:**
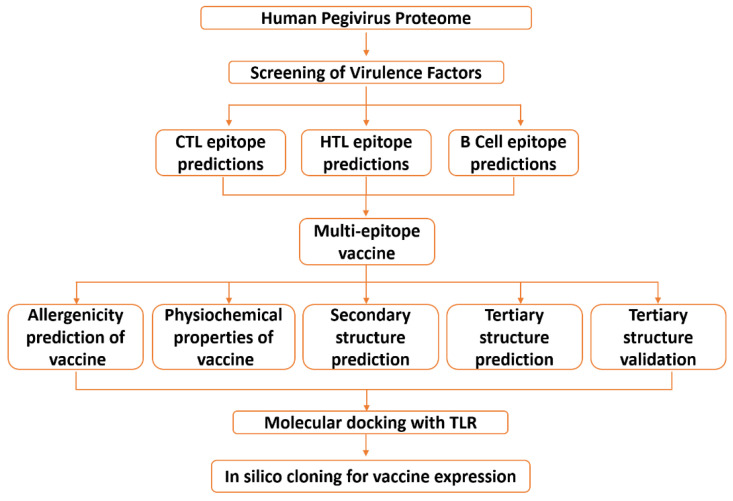
The overall workflow: from proteome retrieval to multi-epitope vaccine design and its validation through in silico cloning and immune simulation.

The proteome for the human Pegivirus was collected in FASTA format from UniprotKB (https://www.uniprot.org/) (accessed on 15 December 2020) and used for further analysis. The workflow for this study is represented in Figure 1.

### 2.2. Data Processing

#### 2.2.1. Mapping Immunogenic Peptides in the Pegivirus Proteome

For prediction of cytotoxic T-lymphocytes, an online web server NetCTL 1.2 (http://www.cbs.dtu.dk/services/NetCTL/) (accessed on 15 December 2020) [25] was used. The key components the server uses for prediction are MHC-I (major histocompatibility complex) binding peptide prediction, transportation efficiency TAP (transport associated with antigen processing), and proteasomal C terminal cleavage. The threshold for CTL (cytotoxic T lymphocytes) epitopes prediction was set to 0.75. HTL (helper T lymphocytes) epitope prediction was performed with the IEDB tool (http://www.iedb.org/) (accessed on 15 December 2020) using the seven reference alleles set provided [26]. Each peptide’s affinity score depends upon the IC50 value. Peptides with IC50 values less than 50 nM have a high binding affinity. Peptides with the IC50 score of less than 500 nM points are intermediates, and peptides with less than 5000 nM point to low binding affinity of the epitopes. The percentile ranks and affinity scores of the peptides are inversely related, meaning the higher the binding affinity, the lower the percentile rank, and vice versa. The affinity of the peptide for each receptor is based on the IC50 score given to each and every predicted epitope. Peptides with higher binding affinity are required to have an IC50 value less than 50 nM; meanwhile, the IC50 score less than 500 nM points to an intermediate, and less than 5000 nM point to the low binding affinity of the epitopes, respectively. The binding affinity and percentile rank of the predicted epitopes are linked inversely: a lower percentile rank is linked with a higher binding affinity, and vice versa. The same server was used for immunogenicity prediction as well. BCPred (http://ailab.ist.psu.edu/ bcpred/) (accessed on 15 December 2020) server was used for this purpose. The server analyzes SVM (Support Vector Machine) by the Kernel Method and uses AAP (amino acid pair) antigenicity for linear B-cell epitope prediction [27].

#### 2.2.2. Multi-Epitope Vaccine Construction and Characterization

A vaccine sequence was developed with the predicted CTL, HTL, and B-cell epitopes. AAY linkers were used for joining CTL epitopes, GPGPG for HTL, and KK (lysine-lysine) for joining B-cell epitopes, respectively [28]. The vaccine sequence was constructed with the final predicted CTL and HTL epitopes filtered through the discussed immunoinformatics strategy. Beta-defensin was added as an adjuvant to the start of the sequence construct [29]. The AllerCatPro server (https://allercatpro.bii.a-star.edu.sg/) (accessed on 16 December 2020) was used to check the allergenicity and classified the sequence as non-allergen at a threshold of 0.001 [30]. VaxiJen server (http://www.ddg-pharmfac.net/vaxijen/VaxiJen/VaxiJen.html) (accessed on 16 December 2020) predicted the antigenic nature of our multi-epitope subunit vaccine. The score predicted was 0.5, while a 0.4 threshold was used for this analysis. The results suggest that our vaccine candidate possesses antigenic properties to provoke an immune response [31]. ProtParam tool (https://web.expasy.org/protparam/) (accessed on 16 December 2020) was employed to calculate nine physicochemical properties, such as molecular weight, theoretical PI, and GRAVY, of the final vaccine construct. In vivo and in vitro half-life was also estimated for the query sequence [32]. The secondary structure of the vaccine construct was predicted via PDBsum (https://www.ebi.ac.uk/pdbsum/) (accessed on 16 December 2020), a feasible online webserver for secondary structure prediction that carries out a complete analysis of protein chains [33]. We also designed a negative control to validate our results.

#### 2.2.3. Structural Modeling, Refinement, and Validation of Vaccine Construct

Tertiary structure prediction of the multi-epitope vaccine construct and the negative construct was performed by Robetta (http://robetta.bakerlab.org/) (accessed on 19 December 2020). Since 2014, the server has been categorized as one of the most precise and consistent servers. It utilizes Continuous Automated Model Evaluation (CAMEO), and every week it calculates up to 20 pre-released PDB targets. The average LDDT (Local Distance Difference Test) score of this server is approximately 69, showing a superposition independent score. It evaluates inter-atomic distances with values ranging from 0 (considered as bad) to 100 (good). PyMOL was used to visualize the 3D structure of the vaccine construct [34]. The tertiary structure of the vaccine construct was refined by Galaxy Refine tools (http://galaxy.seoklab.org/) (accessed on 22 December 2020). The server uses the CASP10 refinement method for side chain reconstruction and repacking of the amino acid sequence, and MD (molecular dynamic) simulation for 3D structure relaxation of the query sequence [35]. ProSA-web (https://prosa.services.came.sbg.ac.at/prosa.php) (accessed on 28 December 2020) and RAMPAGE (http://mordred.bioc.cam.ac.uk/~rapper/rampage.php) (accessed on 28 December 2020) were used for tertiary structure validation [36]. An early score was assigned to the input sequence, and if the sequence was outside the range of native proteins, it more likely indicated errors in the structure of the query protein. For non-bonded interaction, another 3D structure validation tool, ERRAT (http://services.mbi.ucla.edu/ERRAT/) (accessed on 28 December 2020), was used [37]. A Ramachandran plot for the vaccine construct was developed using RAMPAGE [38], which uses the PROCHECK principle for the analysis.

### 2.3. Validation of the Vaccine Construct Immune Potential

#### 2.3.1. Molecular Docking

The vaccine construct was docked with PDB ID: 1ZIW TLR3 (Human Toll-like receptor) using HDOCK server (http://hdock.phys.hust.edu.cn/) (accessed on 2 January 2021). The server uses a hybrid docking strategy and requires an amino acid sequence as input for the analysis, thus distinguishing it from the other servers with the same purpose. It is fast and well recognized for providing experimental information about the protein–protein binding site and small-angle X-ray drip, which can be assimilated during the docking and post-docking processes [39]. Prior to protein–protein docking, the vaccine construct modeled using Robetta was prepared, and the TLR-3 structure was obtained from the protein data bank using PDB ID: 1ZIW. Any heteroatom, water molecules, and other atoms were removed, and the structure was prepared. The negative construct was also tested for interaction with immune receptors.

#### 2.3.2. Molecular Dynamic (MD) Simulation

MD simulations were performed using AMBER 14 package smearing the “tLeap” Amber package. Na ions and hydrogen were added to the system for neutralization. SANDER module of the AMBER package [40] was used in two stages for energy minimization. Using TIP3water box of 12.0 A°, each stage comprised 6000 steps for the removal of constrain atoms in the system. The complexes were then exposed to PMEMD.cuda [41] for MD simulations. For long-term interactions, the SHAKE PME method was used with a non-bond contact cutoff radius of 10 A°. A 100 ns for the vaccine and TLR-3-vaccine complex was carried out, and constant temperature and pressure with isotropic ascending were applied for equilibration of 10,000 ps time. A 2.0 ps time scale was used for the analysis of post-simulation trajectories, and for trajectory sampling CPPTRAJ and PTRAJ in AMBER 14 were applied.

#### 2.3.3. In Silico Cloning and Optimization of Vaccine Protein

To express the multi-epitope subunit vaccine in an appropriate vector, we used the Java codon adaptation tool (JCat tool) [42] for codon optimization and reverse translation. Codon optimization is essential for the expression of vaccine structure in an *E. coli* (strain K12) host, as the usage of the codon is comparatively different in *E. coli* than the native host. Rho-independent transcription, restriction cleavage sites, and prokaryotic ribosome binding sites were eluded through considering three extra options. Java codon adaptation tools provide the output in terms of CAI (codon adaptation index) and %GC content in order to confirm the high level of protein expression. For cloning the final vaccine in *E. coli*, pET-28a (+) vector modification of N and C terminals with XhoI and NdeI restriction sites were performed, respectively. A negative control with no vaccine sequence construct was also generated. Finally, for expression, the prepared optimized sequence, along with the restriction sites, was installed to the pET-28a (+) vector utilizing the SnapGene tool.

#### 2.3.4. Immune Simulation

An agent-based modelling server C-ImmSim (http://www.cbs.dtu.dk/services/C-ImmSim-10.1/) (accessed on 18 January 2021) was used to predict the relationships between the human immune system and the foreign particle, for observing the human immune response to the invading particle. The PSSM method was applied to calculate the production of cytokines as well as other substances such as interferon and antibodies. Furthermore, responses for T helper cell 1 and T helper cell 2 (Th1 and Th2) were also predicted with the default parameter measure of diversity or Simps Index by the server [43].

## 3. Results

### 3.1. Sequence Retrieval and Antigenicity Profiling

The whole polyprotein of Pegivirus was retrieved from the NCBI database and scanned for potential B cell and T cell epitopes to be subsequently used in the design of a multi-epitope peptide vaccine. In the current study, computational modelling and integrated immunoinformatics methodologies were used to develop a multi-epitope vaccine to help the development of immunity against the emerging Pegivirus. Figure 1 represents the overall flow of the work.

### 3.2. Immunogenic CTL, HTL, and B-Cell Epitopes Prediction

Humoral immune response enforcement is a scientifically considerable means to clear up the pathogens by releasing immune agents. T cell, B cell, and helper cells coordinately work to recognize a pathogen and induce long-term immunity. The enforced cell-mediated and adaptive immunity against a particular pathogen is memorized, and the antibodies are stored for long-term responses upon subsequent encounters with the same pathogen. The MHC glycoprotein molecules on the nucleated cell surface play a vital role in presenting peptides from intracellular to cytotoxic T cells, as well as exogenous proteins, in order to cause a rapid immune response to fully eliminate the cell. Thus, the role of CTL is clear in the immune response against a pathogen. Therefore, herein we predicted CTL epitopes in the whole proteome of Pegivirus. A total of 1302 CTL epitopes were predicted, of which only 74 CTL epitopes were predicted to be MHC binders. Seven epitopes were reported to be the strongest binders with favorable immunogenic properties. These include ATDALSTGY_1151-1160_, NSNKTPLLY_1690-1669_, ATHPICWDY_126-135_, LTDTGDVEF_1442-1551_, AVESAMVFY_1595-1604_, YLTNKHSHY_2397-2406_, and HQSESYLKY_273-282_ and were finalized based on antigenicity, C-terminal cleavage, and TAP score. Table 1 lists the selected CTL epitopes and their immune-related predicted features.

Helper T cells, on the other hand, are the most important factors in acquired or adaptive immunity; they assist not only the B-cell in developing and releasing antibodies but also the CTL in killing diseased cells [44]. Upon the activation and presentation on the surface of APCs (antigen-presenting cells) they act as and effector and thus mature into a specific type of HTL required for a specific immune response [45]. To boost up the immune response, HTL epitopes were also finalized on the basis of different parameters using the seven reference alleles and default method by the server. Based on the lowest percentile rank, the HTL epitopes that could induce robust immune enforcement were shortlisted and included ARRGFRMSNNPLSLL_521-535_ (HLA-DRB3*02:02), LVFILSNSSVTTWAN_1970-1984_ (HLA-DRB3*02:02), ASRLRFWLVASAILA_3039-3053_ (HLA-DRB1*07:01), IVLVFILSNSSVTTW_1968-1982_ (HLA-DRB3*02:02), AFLIYILSHPVNAAL_783-797_ (HLA-DRB1*15:01), DGLFPIRHATAALRF_757-771_ (HLA-DRB5*01:01), SVAVVKSMAPYIKET_1328-1342_ (HLA-DRB5*01:01), SRVWVMNNNGGLVCG_1202-1216_ (HLA-DRB3*02:02), KWKCLLNNSNKTPLL_1683-1697_ (HLA-DRB3*02:02), and GLLWQMFVSFPILYS_229-243_ (HLA-DRB1*15:01), as shown in Table 2.

Moreover, mapping B cell epitopes is crucial for immune triggering because the antibodies that bind to epitopes could lead to the activation of five protection mechanisms, which include neutralization, agglutination, complement activation, cell-mediated cytotoxicity directed by antibody coating, and opsonization to augment phagocytosis by tagging antigens with antibodies to augment phagocytosis [46]. The BCPred server effectively mapped 12 linear B-cell epitopes with scores that would help the T-cell and HTL to robustly induce an immunological response and enhance the antigenicity of the multi-epitope subunit vaccine. A list of B-cell epitopes with their position and BCPred predicted scores is given in Table 3.

### 3.3. Structural Prediction of Final Vaccine Construct

**Figure 2:**
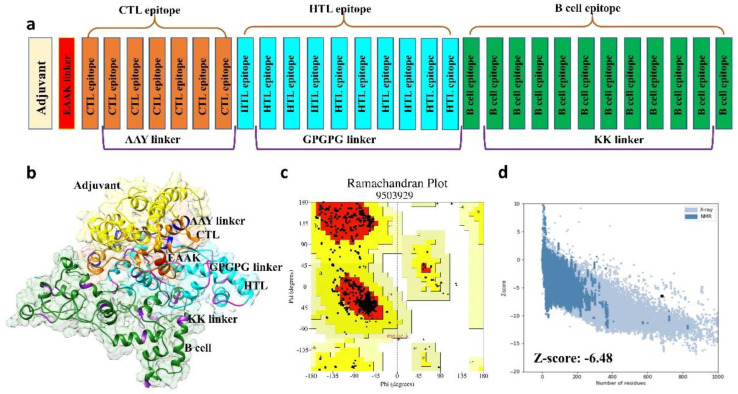
(**a**) Structure of the final multi-epitope vaccine construct and validation of the 3D homology model. (**b**) 3D structure of the vaccine construct provided after homology modeling. CTL, HTL, and B cell epitopes along with linkers are depicted. (**c**) Structure validation of the multi-epitope vaccine by Ramachandran plot. (**d**) ProSA 3D structure validation illustrating Z-score (−6.48).

The vaccine sequence was constructed using the final CTL, HTL, and B-cell epitopes. Using the EAAAK linker, an adjuvant (beta-defensin) was attached to the N-terminal joining by CTL epitopes (Figure 2a). All CTL epitopes were fused with the help of AAY linkers, and HTL epitopes were joined with each other using GPGPG linkers. Further, KK linkers were used for joining B-cell epitopes. The Robetta server initiated five models of the three-dimensional structure of the final vaccine sequence (Figure 2b). The 3D structure of the negative control results shows that for most of the region the structure prediction is not possible, as there was a lack of templates; thus, the best combination of peptides was already selected for the multi-epitope vaccine positive construct. The best model was selected for post structure evaluation.

### 3.4. 3D Structure Validation

The best model initiated by the Robetta server that was selected on the basis of certain factors, such as RMSD value and high GDT-HA score, was used further for 3D structure analysis. Moreover, ProCheck results for the vaccine construct showed 90.6% of amino acids were in the favored region, and 9.4% of amino acids were plotted in allowed regions (Figure 2c). ProSA–web and ERRAT validated the three-dimensional structure of the vaccine construct with the quality factor of 94.1% and Z-score −6.48 by ERRAT and ProSA-web, respectively (Figure 2d).

### 3.5. Prediction of Allergenicity

The AllerCatPro server (https://allercatpro.bii.a-star.edu.sg/cgi-bin/allergy2newblast.pl) (accessed on 25 January 2021) was used to check the allergenicity of the vaccine construct. According to the E-value (threshold 0.001) no evidence of allergenicity was found. On the other hand, the negative vaccine construct was also classified as allergenic.

### 3.6. Antigenicity of the Vaccine Construct

VaxiJen server predicted the antigenic nature of our final multi-epitope subunit vaccine. The score predicted was 0.5, while 0.4 was used as a threshold for this analysis. The results suggest that our vaccine candidate possesses antigenic properties that will help to provoke an immune response. Furthermore, the negative vaccine construct was also classified as antigenic as it possesses antigenic properties. This is a principally acceptable phenomenon, that when any foreign particle enters into the body it is recognized as an antigen. VaxiJen server predicted the antigenic nature of our final multi-epitope subunit negative construct. The score predicted was 0.406, while 0.4 was used as a threshold for this analysis. The results suggest that the negative construct is not highly antigenic and only crosses the baseline value.

### 3.7. Physiochemical Parameters Prediction

**Figure 3:**
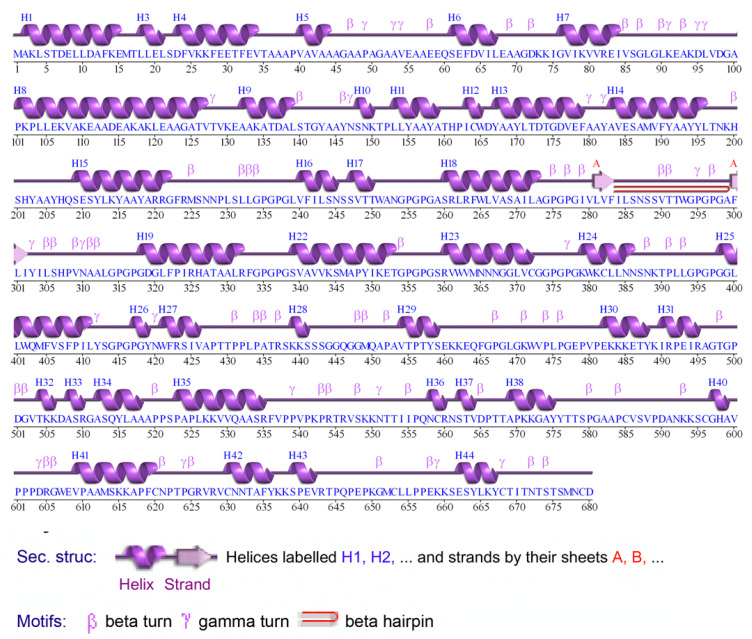
Secondary structure of the predicted vaccine ensemble. The figure shows that helices are interspersed in the vaccine ensemble structure stabilized by beta-sheets and connected by loops.

The ProtParam server was employed to calculate the nine physiochemical properties of the final vaccine construct. The molecular weight of 71.6 kDa and theoretical protrusion index (PI) of 9.32 of the vaccine indicated the basic nature of the vaccine. The estimated in vivo half-life in *E. coli* was >10 h. The stability of the vaccine in the test tube was determined by the instability index, and this value for the final vaccine construct was 38 (the value below 40 is considered stable in nature). The aliphatic index value was 73.69, which indicates the thermostable nature at variable temperatures. The Grand Average Hydropathy value of the vaccine was −0.198, suggesting its hydrophilic nature. The physiochemical properties of the negative control revealed that the construct half-life in *E. coli* was less than 10 h (~2 h), which itself explains that this negative MEVC is not suitable for downstream processing. Thus, this result indicates that this protein is not expressed and purified for vaccine production. Consequently, this shows that our vaccine construct displays excellent properties that are required for immune response triggering and downstream process. On the other hand, no such combination of peptides predicted by different servers exhibited the minimal required feature for the effective vaccine candidate. The secondary structure of the vaccine construct is characterized in Figure 3.

### 3.8. Molecular Docking

**Figure 4:**
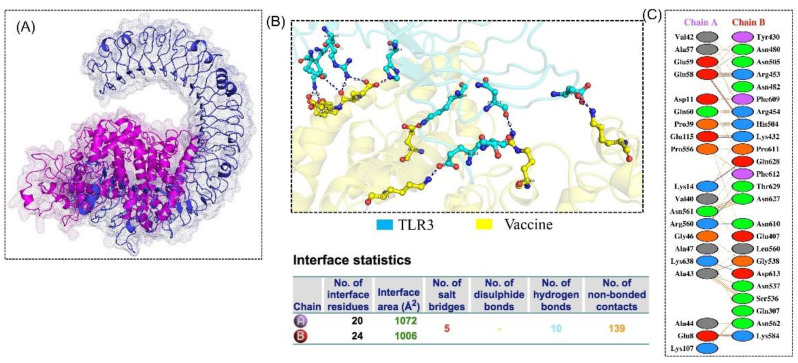
TLR-3-vaccine docked complex. (**A**) The TLR-3(receptor) is shown in blue, while the magenta color represents the multi-epitope subunit vaccine. (**B**)The residues interactions in 3D are shown while (**C**) in the right-side panel shows 2D interactions.

The vaccine construct was docked with human TLR 3 with PDB ID: 1ZIW, in order to observe the immune response. Docking was carried out via the HDOCK server. Only one large-sized cluster with a low energy score was generated from the 30 models, as it showed better interaction between the receptor and ligand. Figure 4 shows chemical interactions and the docked complex confirmation. Molecular docking revealed that the designed vaccine ensemble formed five salt bridges, 10 hydrogen bonds, and 139 non-bonded interactions. Unlike the MEVC-positive construct, the negative construct did not fold well, and the structure was mostly enriched with loops. The binding of the negative construct revealed that, though it is common that docking will predict the interaction, the structure could not occupy the binding site well, and its folding also questions the proper interactions.

### 3.9. Structural Dynamics Features of the Vaccine Ensemble and TLR3 Complex

**Figure 5:**
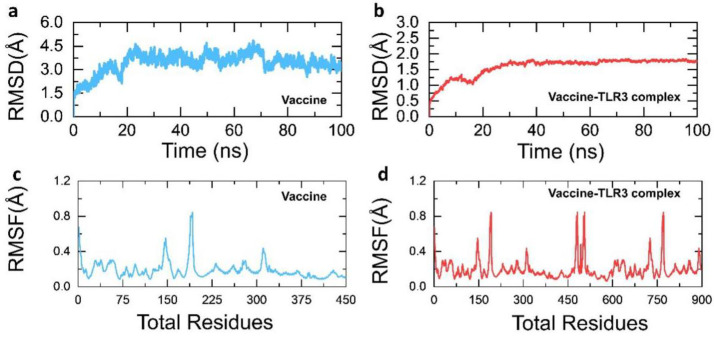
The dynamic properties RMSD(s) and RMSF(s) of the vaccine and vaccine-TLR3 complex. (**a**) show the RMSD of the MEVC, (**b**) RMSD of vaccine-TLR3 complex, (**c**) RMSF of the MEVC while (**d**) show the RMF of vaccine-TLR3 complex.

Molecular dynamics-based stability investigations of the vaccine and vaccine-TLR3 complex are reported to be stable during the simulation, which confirm the stable folding and sustained, robust interaction of the vaccine molecule with the TLR3. The average RMSD (root-mean-square deviation) for the vaccine model was only 3.0 Å, while for the vaccine-TLR3 complex an RMSD of 2.0 Å with minimal convergence was reported, which shows the stable behavior over the 100 ns simulation. In addition, the residual fluctuation was also observed to be minimal in the vaccine model, except for higher fluctuations between 100 and 225 as well as 300 and 325 regions, while in the vaccine-TLR3 complex 100–225, 300–325, 450–500, and 675–775 regions fluctuated more. All the MD results including RMSD and RMSF (root-mean-square fluctuation) are given in Figure 5a–d.

### 3.10. Codon Optimization and In Silico Cloning

**Figure 6:**
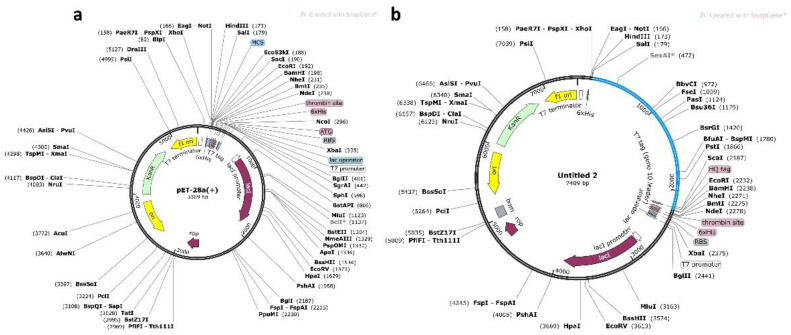
In silico restriction cloning of the final vaccine construct into the pET28a (+) expression vector where the blue part represents the vaccine insert and black circle shows the vector. (**a**) represents the negative control construct while (**b**) represents the vaccine construct inserted into the vector shown as blue.

JCAT tool optimized the vaccine sequence codon usage according to the *E. coli* K12 strain with CAI estimated as 0.96 and GC content 56.24%, showing the better expression level of vaccine construct in *E. coli* (K12 strain). GC contents between 35 and 70% have been reported for better expression of the gene. For the negative construct, the CAI value was reported to be 0.78 only while the GC contents were 55.10%. Lastly, using SnapGene, the adapted vaccine was then cloned into the pET28a (+) plasmid using the same tool with restriction enzymes Xh0I and NdeI. The sites for these enzymes were implanted to both ends, N-terminal and C-terminal, of the optimized vaccine construct and cloned in the pET-28a (+) vector. For instance, a negative control is used in vaccine trials to check the effectiveness or for the purification of vaccine. Considering the role of negative control, a construct was designed here that can be used as an empty and recombinant vector to express the protein and then compare the recombinant protein with the negative control to confirm the purity of protein. The total size of the cloned vector is 7409 bps, as shown in Figure 6a and Figure 5B.

### 3.11. Immune Simulation Analysis

**Figure 7:**
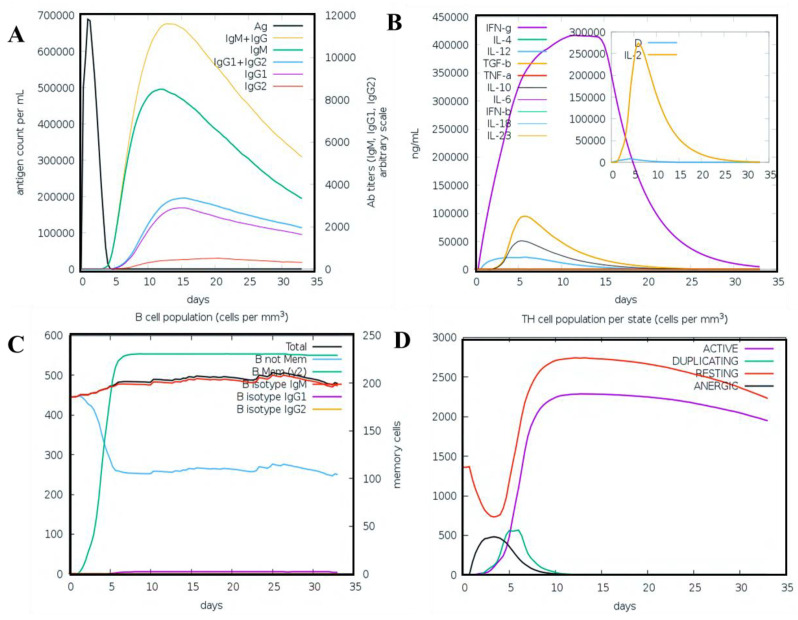
C-ImmSim presentation of an in silico immune simulation with the chimeric peptide. (**A**) Antibodies production (the black vertical lines are antigen). (**B**) Cytokine level (**C**,**D**) shows the B and T-cell populations, respectively.

After the injection of multiple doses of the antigen, the response of the human immune system was perceived using a computational immune response modelling approach. The immune response was significantly triggered, and the antibody titer was very high after the injection (Figure 7A). It was observed that the combined IgM + IgG titer remained at 700,000 xx/mL; the IgM titer alone was computed to be around 55,000 xx/mL while Ig1 was reported as 15,000 xx/mL. Interleukin (IL) and cytokine responses were also analyzed (Figure 7B). The IFN-g and IL-2 titers were significantly high, showing a consistent and robust immune-triggering response upon injection. The development of memory cells and the cellular immune system response to pathogen identification at re-encounter was also very strong. The T cell population was reported as >1150 cells/mm^3^ (Figure 7C). A maximum concentration of 375 cells/mm^3^ for the phagocytic natural killer cell population was reported (Figure 7D). Likewise, dendritic cells and the phagocytic macrophage population were reported to be 200 cell/mm^3^, respectively. Comparatively the negative control results shown in the Supplementary Materials show that the antigen titer was given as high as 4.5 × 10^7^, but still the response could not be provoked efficiently. The antigen remained at a higher load for the first 20 days and for different injections. This shows that the positive MEVC triggered a more robust immune response compared to the negative construct. Hence, these results confirm that our vaccine candidate effectively triggers an immune response.

## 4. Discussion

Vaccination is an effective way to control infectious diseases caused by microbial pathogens. In this regard, mapping antigenic epitopes on the virulent proteins helps in designing vaccine candidates. Early and rational design of vaccine candidates for outbreaks can restrain infections; however, compared to the advanced computational vaccine designs, the conventional approaches suffer many problems. The most important advantages of the in silico-based design include less time consumption, more economical, more stable, and more antigenic than the conventionally methods. Integration of highly immunogenic T cell, B cell, and HTL epitopes from the proteome of a pathogen provides a better solution for the treatment. Considering the advantages of immunoinformatics approaches, many vaccine candidates have been designed and tested experimentally [24,47,48,49]. Pegivirus is a human pathogen that causes several disorders in humans. To date, no effective vaccine candidates are available for the treatment of any potential outbreak caused by this virus. Thus, to manage any potential future outbreak, computational data analyses (i.e., vaccine designing, drug designing, understanding the molecular pathogenesis and other features) may help to address health-related issues in a timely manner [50]. Thus, computational vaccinology methods were here employed to map the effective immunogenic peptides in the proteome of Pegivirus and to design MEVC that is safe, non-toxic, non-allergenic, and antigenic for the treatment of infections caused by Pegivirus.

The current study focused on the use of immunoinformatics and molecular modelling methods to design an effective vaccine candidate against Pegivirus infections. Using online tools, B cell, T cell, and HTL epitopes were successfully mapped on the proteome of Pegivirus. Similar approaches have already been used by previous studies to obtain antigenic, non-allergenic epitopes for different pathogens [24,49]. Herein, we successfully mapped 1304 CTL epitopes, among which only 74 were characterized as MHC binders, while only 10 HTL epitopes were reported to exhibit the lowest percentile rank. Moreover, BCPred only shortlisted 12 B cell epitopes with good total scores. This approach of combining epitopes that are good MHC binders and have the lowest percentile rank and the highest B cell epitope score is widely used in designing MEVC [24,48,49]. More important, the physiochemical properties of the MEVC also determine the fate of the designed vaccine candidate. It has been reported that the GC contents, mass, GRAVY, and pI also help in obtaining suitable vaccine candidates. Our predicted physiochemical properties reflect the best properties exhibited by our designed MEVC, which are in accordance with the previously reported literature [51,52]. Furthermore, the antigenicity and allergenicity scores of the vaccine candidate enabled the downstream processing to unveil the immune receptor interaction and expression. The designed candidate vaccine was classified as non-allergenic and antigenic in nature by the webservers. Afterwards, the 3D structure and interaction with the host immune receptor further revealed the efficiency of the vaccine candidate. The interaction of the MEVC was reported to be more robust. The dynamic stability of the MEVC investigated through MD simulation further confirmed its stable folding and interaction.

Jcat software was used for codon optimization in order to improve the transcription and translation effectiveness of the vaccine protein in the K12 strain of *E. coli*. The reverse-translated optimised sequence codon adaptation index value (CAI) and GC content are linked to protein expression. The CAI and GC values suggested that our vaccine protein was highly expressed, while the negative control was also checked to validate the expression of our MEVC only in the positive vector. Additionally, immune simulation further revealed that upon injection, a robust immune response was triggered. All these features were also calculated for the negative construct, and the results are shown in Supplementary Materials. Conclusively, the current study employed a widely used pipeline to design the MEVC; however, the only limitation of the current findings is the experimental testing and validation of the final vaccine candidate, which would pave the way towards clinical use.

## 5. Conclusions

In the current study, through different immunoinformatics tools, a safe and stable multi-epitope-subunit vaccine was designed against *Pegivirus.* Various immunoinformatics tools were employed for the selection of suitable proteins for vaccine design. Online servers were used for the prediction of B cell and T cell epitopes. To construct a vaccine, CTL and HTL epitopes were joined together by using specific linkers and adjuvants. Antigenicity and allergenicity, followed by physiochemical properties, were also checked and confirmed that the vaccine is safe and stable. Molecular docking of the vaccine was also carried out to ensure the interaction between human and TLR-3. Codon optimization and reverse translation were performed. Lastly, the vaccine was in silico cloned in the *E. coli* pET28a+ plasmid to ensure its effective expression and stability. The vaccine designed in this scientific study needs experimental validation to support its potential and safety. The present study will aid in the management of *Pegivirus* infections.

## Figures and Tables

**Table 1:** List of final CTL epitopes selected on the basis of C-terminal cleavage, TAP score, and antigenicity.

Residue No	Peptide Sequence	MHC Binding Affinity	Rescale Binding Affinity	C-terminal Cleavage Affinity	Transport Affinity	Prediction Score	MHC-I Binding
1511	ATDALSTGY	0.7930	3.3668	0.9637	2.9180	3.6573	YES
1690	NSNKTPLLY	0.6869	2.9163	0.9593	3.0490	3.2126	YES
126	ATHPICWDY	0.5329	2.2626	0.9680	3.2180	2.5687	YES
1442	LTDTGDVEF	0.5175	2.1971	0.9251	2.3890	2.4553	YES
1595	AVESAMVFY	0.5148	2.1858	0.4948	3.0140	2.4108	YES
2397	YLTNKHSHY	0.4236	1.7984	0.9649	2.9890	2.0925	YES
273	HQSESYLKY	0.4093	1.7378	0.9614	3.0060	2.0323	YES

**Table 2:** Helper T-cell epitopes for selected proteins of human Pegivirus using the IEDB MHC-II module.

S. No	Allele	Start	End	Peptide Sequence	Method	Percentile Rank
1	HLA-DRB3*02:02	521	535	ARRGFRMSNNPLSLL	NetMHCIIpan	0.01
2	HLA-DRB3*02:02	1970	1984	LVFILSNSSVTTWAN	NetMHCIIpan	0.01
3	HLA-DRB1*07:01	3039	3053	ASRLRFWLVASAILA	Consensus (comb.lib./smm/nn)	0.02
4	HLA-DRB3*02:02	1968	1982	IVLVFILSNSSVTTW	NetMHCIIpan	0.02
5	HLA-DRB1*15:01	783	797	AFLIYILSHPVNAAL	Consensus (smm/nn/sturniolo)	0.13
6	HLA-DRB5*01:01	757	771	DGLFPIRHATAALRF	Consensus (smm/nn/sturniolo)	0.13
7	HLA-DRB5*01:01	1328	1342	SVAVVKSMAPYIKET	Consensus (smm/nn/sturniolo)	0.14
8	HLA-DRB3*02:02	1202	1216	SRVWVMNNNGGLVCG	NetMHCIIpan	0.19
9	HLA-DRB3*02:02	1683	1697	KWKCLLNNSNKTPLL	NetMHCIIpan	0.2
10	HLA-DRB1*15:01	229	243	GLLWQMFVSFPILYS	Consensus (smm/nn/sturniolo)	0.21

**Table 3:** Table of linear B-cell epitopes predicted by the BCPred server.

S. No	Position	Epitope	Score
1	2494	YNWFRSIVAPTTPPLPATRS	1
2	1277	SSSGGQGGMQAPAVTPTYSE	1
3	494	EQFGPGLGKWVPLPGEPVPE	1
4	1340	KETYKIRPEIRAGTGPDGVT	1
5	1811	DASRGASQYLAAAPPSPAPL	1
6	2318	VVQAASRFVPPVPKPRTRVS	0.999
7	369	NTTIIPQNCRNSTVDPTTAP	0.999
8	1572	GAYYTTSPGAAPCVSVPDAN	0.999
9	663	SCGHAVPPPDRGWEVPAAMS	0.998
10	545	APFCNPTPGRVRVCNNTAFY	0.998
11	3000	SPEVRTPQPEPKGMCLLPPE	0.997
12	275	SESYLKYCTITNTSTSMNCD	0.996

## Data Availability

All the data will be provided on reasonable demand.

## Supplementary Materials

The following are available online at https://www.mdpi.com/article/10.3390/vaccines9080818/s1, Figure S1: Molecular Docking, secondary structure and Immune simulation results for the negative vaccine construct. (A) Docking results of the negative vaccine construct, which reflects that the vaccine interacts with correct binding site, but most of the residues are modelled as loop regions, thus questioning the validity. (B Interaction pattern, (C) secondary structural elements distribution, and (D) antibody titer production over time.
