# Supplementary material for: Towards an Ensemble Vaccine against the Pegivirus Using Computational Modelling Approaches and Its Validation through In Silico Cloning and Immune Simulation
Source: Vaccines (Basel). 2021 Jul 23;9(8):818. doi: 10.3390/vaccines9080818 (PMC8402528; doi:10.3390/vaccines9080818)
Supplement: Supplementary file 1 [file vaccines-09-00818-s001.zip › vaccines-1272586-supplementary.pdf]

Supplementary Materials

Towards an ensemble vaccine against the Pegivirus using computational modelling approaches and its validation through *In silico* cloning and immune simulation

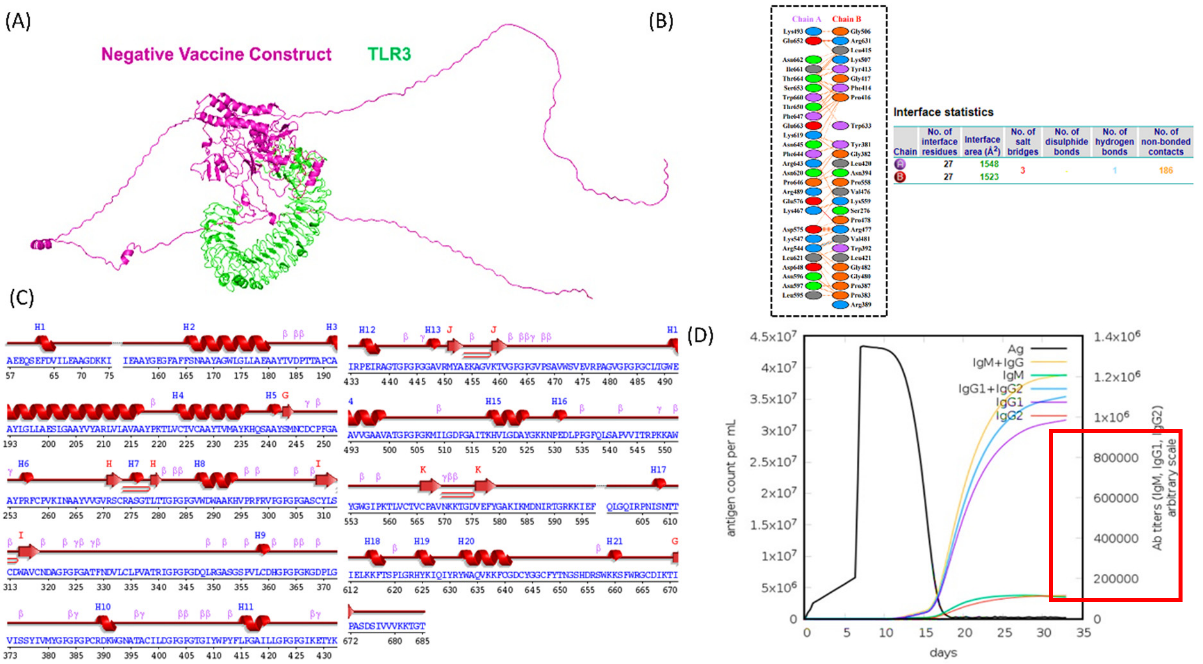

Figure S1: Molecular Docking, secondary structure and Immune simulation results for the negative vaccine construct. (A) show the docking results of the negative vaccine construct which reflects that though the vaccine interacts with correct binding site but most of the residues are modelled as loop region thus question the validity. **(B)** show the interaction pattern, **(C)** shows the secondary structural elements distribution while **(D)** display the antibodies titer production over the days.

>Negative Control Vaccine Sequence

MAKLSTDELLDAFKEMTLLELSDFVKKFEETFEVTAAAPVAVAAAGAAPAGAAVEAAEEQSEFDVI
LEAAGDKKIGVIKVVREIVSGLGLKEAKDLVDGAPKPLLEKVAKEAADEAKAKLEAAGATVTVKE
AAKCSIRSKTEGAAYRSKTEGRIEAAYGEGFAFFSNAAYAGWLGLLAEAAYTVDPTTAPCAAYLGL
LAESLGAAYVYARLVLAVAAYPKTLVCTVCAAYTVMAYKHQSAAYSMNCDCPFGAAYPRFCPVK
INAAYVVGVRSCRASGTLTTGPGPGVWDWAAKHVPRFRVPGPGPGASCYLSCDWAVCNDAGPG
PGATFNDVLCLPVATRIGPGPGDQLRGASGSPVLCDHGPGPGGAVRMYAEKAGVKTVGPGPGVPS
AVWSVEVRPAGVGPGPGCLTGWEAVVGAAVATGPGPGKMILGDPGAITKHVLGDAYGKKNPED
LPPGFQLSAPVVITRPKKAWYGWGIPKTLVCTVCPAVNKKTGDVEFYGAKIKMDNIRTGRKKIEFV
QLGQIRPNISNTTIELKKFTSPLGRHYKIQIYRYWAQVKKFCGDCYGGCFYTNGSHDRSWKKSFWR
GCDIKTIPASDSIVVVKK
